# Dual-Use Research and Technological Diffusion: Reconsidering the Bioterrorism Threat Spectrum

**DOI:** 10.1371/journal.ppat.1001253

**Published:** 2011-01-13

**Authors:** Jonathan E. Suk, Anna Zmorzynska, Iris Hunger, Walter Biederbick, Julia Sasse, Heinrich Maidhof, Jan C. Semenza

**Affiliations:** 1 Scientific Advice Unit, European Centre for Disease Prevention and Control, Stockholm, Sweden; 2 Research Group for Biological Arms Control, University of Hamburg, Hamburg, Germany; 3 Federal Information Center for Biological Security, Robert Koch Institute, Berlin, Germany; The Fox Chase Cancer Center, United States of America

The global security community continues to view a potential bioterrorist event with concern. Kofi Annan, former Secretary General of the United Nations, stated “the most important under-addressed threat relating to terrorism…is that of terrorists using a biological weapon” [1]. The European Commission believes that biological weapons “may have particular attractions for terrorists” [2]. The United States Commission on the Prevention of Weapons of Mass Destruction Proliferation and Terrorism believes it is very likely that a weapon of mass destruction will be used in a terrorist attack by the end of 2013, and that an attack with a biological weapon is more likely than one with a nuclear weapon [3].

There is good reason for concern. Infectious diseases elicit instinctive fears that some terrorist organizations appear to have the intent to exploit [4]. The 2001 anthrax attacks in the United States, believed to have been caused by a single actor [5], were a keen reminder of the ability of bioterrorism to cause death and societal disruption. Such concerns have been linked to the rapid progress in life science research. The most advanced techniques 20 years ago are today routine (and some, like DNA synthesis, are also much cheaper [6]), while new fields, notably synthetic biology [7], [8], have opened frontiers previously inconceivable. Furthermore, expertise in life science research is globally dispersed, and methodologies for synthesizing and/or altering the virulence of pathogens in the laboratory have already been published in high-profile scientific journals. Activities that have garnered substantial attention include chemically synthesizing the poliovirus [9] and the ΦX174 bacteriophage [10], demonstrating the importance of a variola virus gene for its virulence [11], and reconstituting the 1918 influenza virus [12]. Each has been classified as dual use research of concern (DURC), which is defined by the US National Science Advisory Board for Biosecurity (NSABB) as “research that, based on current understanding, can be reasonably anticipated to provide knowledge, products, or technologies that could be directly misapplied by others” [13].

DURC creates a tension between freedom of research and national security [14]–[17]. As security communities have pushed for tighter oversight of research, scientific communities have been quick to grasp that certain biosecurity regulations, such as export controls [18] or visa controls for foreign scientists [19], run the risk of being inadvertently disruptive [20]–[24]. Members of the US NSABB have even argued that the inhibition of life science research could be considered a threat to national security and public health in and of itself [25]. Yet as concerns the rationale for biosecurity controls, the scientific community has been generally muted. Although this may be related to the secrecy surrounding intelligence about terrorist organizations, classified snippets of information should not have priority over expert technical input. Ceding the debate to the security community could lead to inaccurate threat assessments and the adoption of inappropriate biosecurity control measures.

The European Centre for Disease Prevention and Control (ECDC) was established in 2005 with the mandate to strengthen Europe's defenses against infectious diseases through developing European Union–wide surveillance networks and early warning systems, coordinating scientific studies, and identifying emerging health threats [26]. As a part of ECDC efforts to evaluate potential bioterrorism threats, we reviewed 27 assessments (published between 1997 and 2008) that address the links between life science research and bioterrorism with the objective of identifying DURC relevant for public health (Text S1). The focus of the review was limited to the application of DURC by terrorist organizations and it did not consider state-sponsored biological weapons programs.

The 27 assessments were selected based upon a literature review and interviews with a panel of international experts. Collectively, the 27 assessments explicitly cite a wide range of DURC activities. Based upon these, we conducted a threat assessment during an expert workshop. The purpose of this threat assessment was to identify those DURC activities that would be the most easily deployed by bioterrorists. The key parameters for this assessment were the level of expertise required for conducting any given DURC activity and the level of equipment required to conduct the work. In the threat assessment, an estimated threat level was calculated for each DURC activity by giving a score ranging from 1 (high threshold) to 3 (low threshold) for both parameters, and then multiplying these scores to yield the final threat, which could be 1, 2, 3, 4, 6, or 9. Higher scores indicate a higher likelihood of success if they were to be undertaken by bioterrorists (Text S1).

The overall ranking provides an indication of the threat spectrum related to the ability of bioterrorists to exploit life science research (Table 1), and it suggests that “low tech” activities may be especially attractive to bioterrorists. This opposes the tendency of biosecurity discussions to be rather more focused on “high tech” research: typically, the potential negative consequences of research falling into the wrong hands are accentuated while the likelihood of this occurring is inadequately considered. Is the availability of material, methodologies, and high-level expertise, none of which should be taken for granted, even adequate for the development of a sophisticated bioweapon? Technology is much more than the sum of its material and informational aspects. Social contingencies and tacit knowledge, serendipity and unpredictability, institutional memory, and many other factors are essential to the successful design and deployment of any given technology, including (if not especially) biological weapons [27], [28]. Interviews with the Wimmer group about the poliovirus synthesis [9], for example, highlight that replicating the experiment is a very challenging and time-consuming procedure even for virologists familiar with the experimental system [29]. It is not obvious that extrapolating the methods from this work for other purposes—or to another laboratory—would have been successful. The challenge is surely even greater when resource, time, or other constraints (such as the need to be clandestine) are involved.

**Table 1:** Threat assessment for research areas of concern.

	Expertise ThresholdLow – (3)Medium – (2)High – (1)	Equipment ThresholdLow – (3)Medium – (2)High – (1)	Threat Level
Enhance the dissemination of a biological agent by contamination of food or water supplies late in a distribution chain	3	3	9
Increase the environmental stability of a biological agent by mechanical means, e.g., microencapsulation	2	2	4
Confer resistance to therapeutically useful antibiotics or antiviral agents	2	2	4
Facilitate the production of biological agents	2	2	4
Enhance the dissemination of a biological agent by contamination of food or water supplies early in a distribution chain	3	1	3
Enhance the dissemination of a biological agent as powder or aerosol	1	2	2
Synthetic creation of viruses	2	1	2
Render a vaccine ineffective	1	1	1
Enhance the virulence of a biological agent	1	1	1
Increase the transmissibility of a biological agent	1	1	1
Enhance the infectivity of a biological agent	1	1	1
Alter the host range of a biological agent	1	1	1
Render a non-pathogenic biological agent virulent	1	1	1
Insertion of virulence factors	1	1	1
Enhance the resistance of a biological agent to host immunological defence	1	1	1
Insertion of host genes into a biological agent to alter the immune or neural response	1	1	1
Generate a novel pathogen	1	1	1
Increase the environmental stability of a biological agent by genetic modification	1	1	1
Enable the evasion of diagnostic or detection modalities	1	1	1
Targeting materials to specific locations in the body	1	1	1

Calculated according to the formula total threat  =  (expertise threshold) × (equipment threshold), this table presents individual DURC activities according to the ease with which a terrorist organization could be expected to replicate the work, based on expertise and equipment thresholds. The highest threat level comes from DURC activities that were deemed to require overcoming only low expertise and low equipment thresholds (such as contaminating a food or water source with an unaltered pathogen). Conversely, the lowest threat comes from highly sophisticated DURC activities that would need to overcome high equipment and expertise thresholds, such as those that would be required to substantially alter the genetic nature of a pathogen.

The recent history of bioterrorism also suggests that more attention should be allotted to low tech threats [30]. An extensive review of biocrimes in the 20th century argued that although bioterrorists might acquire some capabilities, there is “reason to doubt the ease with which such groups could cause mass casualties” [31]. Aum Shinrikyo, for example, was not successful in procuring, producing, or dispersing anthrax and botulinum toxin in the 1990s, while Al Qaeda is believed to have failed to obtain and work with pathogens by the early 2000s [32], and this likely remains the case. In comparison, the contamination of food and water, and direct injection/application of a pathogen, all have much lower technical hurdles and might be expected to be rather more successfully deployed [31]. The best-known example is the contamination of salad bars with *Salmonella* by the Rajneeshee cult in 1984, which led to roughly 751 illnesses and 45 hospitalizations [33]. It remains the only known incident in which a terrorist organization, rather than an individual, deployed a biological agent in the US [31].

We do not suggest that high tech bioterrorism threats do not exist—rather, that their likelihoods should be re-evaluated. Biosecurity policy discussions could gain more nuance and credibility by adopting more sophisticated notions about the challenges inherent in conducting and replicating advanced research. The life sciences community has an obvious self-interest in this, and might best achieve it by emphasizing the oft-unacknowledged factors inherent to successful high tech research, including those related to social contingencies and tacit knowledge. Thus far, when life scientists have entered the fray, they have tended to reinforce the “high-tech” perspective, even if their objectives have been to argue against strict biosecurity controls and/or to encourage the life sciences to engage in debates about the risks and benefits of its research [34]–[36].

Many agree about the importance of threat mitigation measures that prepare for the eventuality of a bioterrorism attack, irrespective of its source [37], [38]. Examples include encouraging the development of diagnostics, vaccines, and therapeutics, as well as empowering public health agencies to strengthen defenses against communicable diseases. Such approaches have the additional advantage that they take the broadest possible view of the threat spectrum by also preparing for attacks by the most successful “bioterrorists” of all, nature and globalization, which have led to the emergence of numerous new communicable diseases in recent years [39]–[41]. A focus on strengthening global health security has been put forward by the Obama administration [42] and the European Commission [38], and has also gained prominence in fora such as the Biological and Toxin Weapons Convention [43]. Public health, too, is dual use: it can be leveraged to counter natural and intentional disease outbreaks.

## Supporting Information

Text S1 Dual-use assessments reviewed in this study (in reverse chronological order).
(0.05 MB PDF)
