# Supplementary material for: Dual-Use Research and Technological Diffusion: Reconsidering the Bioterrorism Threat Spectrum
Source: PLoS Pathog. 2011 Jan 13;7(1):e1001253. doi: 10.1371/journal.ppat.1001253 (PMC3020926; doi:10.1371/journal.ppat.1001253)
Supplement: Text S1 — Dual-use assessments reviewed in this study (in reverse chronological order). (0.05 MB PDF) [file ppat.1001253.s001.pdf]

Text S1.

Identification of dual-use publications

27 assessments, dating from 1997 to 2008, were used in the project. These assessments were identified and selected through a literature search, grey literature, and interviews with biosecurity experts. Most of the assessments reviewed in this paper were authored by U.S. (8) or European (13) groups. In order to ensure, that non-U.S. and non-European views were not missed, experts in the leading biotechnology countries in all the geographical sub-regions as defined by the UN were contacted and asked for relevant assessments as follows: Australia, Brazil, Cuba, Egypt, India, Israel, Japan, Kenya, Mexico, Nigeria, Russia, Singapore and South Africa. The complete list of the assessments reviewed in this study is listed below, in reverse chronological order:

Molas-Gallart, J; Perry Robinson J P Assessment of dual-use technologies in the context of European security and defence. Final report for the Scientific and Technological Options Assessment (STOA), European Parliament. Brighton: SPRU, 1997, 58p.

Fraser, C.M. & Dando, M. R. Genomics and the future of biological weapons: the need for preventive action by the biomedical community. Nature genetics, Vol 29: 253-256. (2001).

Zilinskas, R.A., J.B. Tucker, Limiting the Contribution of the Open Scientific Literature to the Biological Weapons Threat, Journal of Homeland Security (December 2002).

Do no harm: reducing the potential for the misuse of lifescience research-Report of a Royal Society - Wellcome Trust meeting on Oct. 2004.

Biotechnological Research in an Age of Terrorism-- National Research Council, National Academies of Science (2004).

McLeish C., Nightingale P. The Impact of Dual use Controls on UK Science: Results From a Pilot Study, SPRU Electronic Working paper Series, Paper 132, April 2005.

WHO- Life Science Research: Opportunities and Risks for Public Health. 2005.

Managing risks of misuse associated with grant funding activities A joint BBSRC, MRC and Wellcome Trust policy statement, Sept.2005.

Roffey, R., From bio threat reduction to cooperation in biological proliferation prevention, Background paper 4, Conference on Strengthening European Action on WMD Non-proliferation and Disarmament: How Can Community Instruments Contribute?, Dec. 2005.

Maurer S.M. et al., From Understanding to Action: Community-Based Options for Improving Safety and Security in Synthetic Biology, University of California, Berkeley, Goldman School of Public Policy, April 2006.

National Science Advisory Board For Biosecurity, NSABB Draft Guidance Documents; July 2006.

Campbell P. Empowerment and restraint in scientific communication: New developments make it easier to share information, but more difficult to deal with dual-use biology.EMBO Rep. 2006 July; 7(SI): p.18–S22.

WHO, Biorisk Management- Laboratory Biosecurity Guidance, September 2006.

National Science Advisory Board For Biosecurity; Addressing Biosecurity Concerns Related to the Synthesis of Selected Agents; December 2006.

Atlas, R.M., M. Dando, The Dual-Use Dilemma for the Life Sciences: Perspectives, Conundrums, and Global Solutions, Biosecurity and Bioterrorism: Biodefense Strategy, Practice, and Science Bd. 4 Nr. 3 (2006).

Institute of Medicine &National Research Council- Globalization, Biosecurity, and the future of the life sciences (2006).

Miller, S., M. Selgelid, Ethical and Philosophical Consideration of the Dual-Use Dilemma in the Biological Sciences, Canberra: Centre for Applied Philosophy and Public Ethics, Australian National University and Charles Sturt University, 2006.

Steinbruner et al., Controlling Dangerous Pathogens Project, Center for International and Security Studies at Maryland, March 2007.

Development of a Threat Assessment Framework Applicable to Dual Use Biotechnology Lyle Makosky, Eric Stephen, Defence R&D Canada. April 2007

NSABB - Proposed Framework for the Oversight of Dual Use Life Sciences Research: Strategies for Minimizing the Potential Misuse of Research Information, June 2007.

Biotechnological Research in an Age of Terrorism – The Israel Academy of Science and Humanities and the Israel National Security Council, July 2007.

Kuhlau, F., Countering Bio-threats, EU Instruments for Managing Biological Materials, Technology and Knowledge, SIPRI Policy paper No. 19, August 2007.

Garfinkel M. S. et al., Synthetic Genomics - Options for Governance, CSIS Reports, October 2007.

Commission of the European Communities (2007). Green Paper on Bio-preparedness.

OECD Best Practice Guidelines on Biosecurity for Biological resource Centres (BRCs)- 2007.

Atlas RM, Toward global harmonization for control of dual-use biothreat agents, Science and Public Policy, Volume 35, Number 1, February 2008 , pp. 21-27(7).

Brian Rappert, The benefits, risks, and threats of biotechnology Science and Public Policy, 35(1), February 2008, pp. 37–43.

Identifying dual use research of concern

Each of the 27 assessments was analysed to provide information on the areas of research considered to be of special security concern. Of the 27 assessments identified, 22 refer to specific research activities that could be of concern. Taken together, these 22 assessments list 53 different types of DURC activities. The 53 activities can be categorized broadly in research with dangerous results such as enhancing virulence, and potentially dangerous technologies such as DNA synthesis.

The list was reduced from 53 items to 20 items for inclusion in the threat assessment. Key criterion for inclusion in the shortlist was that the activity would yield directly weapons-usable results. Moreover, a number of the 53 DURC areas were too broad to be useful in a threat assessment, such as genetic engineering or nanotechnology.

Ranking research areas of concern

Each of the 20 activities was assessed according to the threat the activity would pose in the hands of terrorists. The threat assessment looked exclusively at the capabilities of terrorists, guided by a simple question: how likely would it be that the activity/knowledge in question could be applied by a bioterrorist to prepare a biological weapon? The threat assessment was conducted by a group of 13 practising life scientists and public health experts from Germany during a workshop in October 2008. For each of the 20 activities the thresholds to be overcome in terms of expertise and equipment were assessed by the experts in a consensus-oriented discussion according to the following criteria:

Expertise threshold

Low ("undergraduate level") – resulting threat level high (3)
Moderate ("graduate level") – resulting threat level medium (2)
High ("year-long specialized experience") – resulting threat level low (1)

Equipment threshold

Low ("kitchen") – resulting threat level high (3)
Moderate ("diagnostic laboratory") – resulting threat level medium (2)
High ("specialized laboratory") – resulting threat level low (1)

The estimated potential threat level was calculated for each DURC activity by giving a score ranging from 1 (high threshold and thus low threat level) to 3 (low threshold and thus high threat level) for both parameters. These scores were multiplied to yield the final score, which could be 1, 2, 3, 4, 6 or 9. Higher scores indicate a higher likelihood of success if they were to be undertaken by bioterrorists. The final ranking is presented in Table 1 in the Manuscript.
